# Pufferfish Saxitoxin and Tetrodotoxin Binding Protein (PSTBP) Analogues in the Blood Plasma of the Pufferfish *Arothron nigropunctatus*, *A. hispidus*, *A. manilensis*, and *Chelonodon patoca*

**DOI:** 10.3390/md16070224

**Published:** 2018-06-29

**Authors:** Mari Yotsu-Yamashita, Yuuma Nagaoka, Koji Muramoto, Yuko Cho, Keiichi Konoki

**Affiliations:** 1 Graduate School of Agricultural Science, Tohoku University, 468-1 Aramaki-Aza-Aoba, Aoba-ku, Sendai 980-8572, Japan; uma.swimfree200@gmail.com (Y.N.); yuko.cho.a4@tohoku.ac.jp (Y.C.); konoki@m.tohoku.ac.jp (K.K.); 2 Professor emeritus, Graduate School of Life Sciences, Tohoku University, 2-1-1 Katahira, Aoba-ku, Sendai 980-8577, Japan; koji.muramoto.d5@tohoku.ac.jp

**Keywords:** tetrodotoxin, saxitoxin, pufferfish, PSTBP, *Arothron*, *Cheonodon*, TBT-bp2

## Abstract

Pufferfish saxitoxin and tetrodotoxin (TTX) binding protein (PSTBP) is a glycoprotein that we previously isolated from the blood plasma of the pufferfish *Takifugu pardalis*; this protein was also detected in seven species of the genus *Takifugu*. We proposed that PSTBP is a carrier protein for TTX in pufferfish; however, PSTBP had not yet been found in genera other than *Takifugu*. In this study, we investigated the presence of PSTBP-like proteins in the toxic pufferfish *Arothron nigropunctatus*, *A. hispidus*, *A. manilensis*, and *Chelonodon patoca*. On the basis of ultrafiltration experiments, TTX was found to be present and partially bound to proteins in the plasma of these pufferfish, and Western blot analyses with anti-PSTBP antibody revealed one or two bands per species. The observed decreases in molecular mass following deglycosylation with glycopeptidase F suggest that these positive proteins are glycoproteins. The molecular masses of the deglycosylated proteins detected in the three *Arothron* species were larger than that of PSTBP in the genus *Takifugu*, whereas the two bands detected in *C. patoca* had molecular masses similar to that of tributyltin-binding protein-2 (TBT-bp2). The *N*-terminal amino acid sequences of 23–29 residues of these detected proteins were all homologous with those of PSTBP and TBT-bp2.

## 1. Introduction

Pufferfish saxitoxin (STX) and tetrodotoxin (TTX) binding protein (PSTBP) is a dimeric glycoprotein with a molecular mass of 208 kDa that we previously isolated from the blood plasma of the pufferfish *Takifugu pardalis* [1]. This protein is homologous with the TTX-binding protein identified in the blood plasma of *T. alboplumbeus* (former name, *T. niphobles*) by Matsui et al. [2]. The PSTBP monomer is composed of a 42 kDa protein and a 62 kDa *N*-glycan and forms a non-covalent dimer. Two highly homologous cDNA-coded proteins, namely PSTBP1 and PSTBP2, were identified during analysis of the cDNA coding of PSTBP [1]. TTX and STX competitively bind to PSTBP at neutral pH, and the toxins are released from PSTBP under acidic conditions [1,3]. We prepared a polyclonal antibody against unglycosylated PSTBP1 expressed in *Escherichia coli* [4]. Western blot analyses using this antibody revealed that the blood plasma of all seven species tested in the genus *Takifugu* have one or two proteins homologous with PSTBP, with the size in the 105–140 kDa range, whereas in the glycopeptidase-F treated plasma of these species, proteins approximately 43 kDa in size were commonly detected [4]. We also reported the tissue distribution of PSTBP in *T. pardalis* by immunohistochemical methods [5]. Based on these results, we proposed that PSTBP is a carrier protein of TTX and STX in pufferfish plasma. Oba et al. [6] reported that tributyltin-binding protein type 2 (TBT-bp2) isolated from the plasma of Japanese flounder *Paralichthys olivaceus* is highly homologous with PSTBP. The amino acid sequence of the PSTBP from *T. pardalis* showed a 47% identity to that of TBT-bp2 [6,7]. PSTBP monomers can be thought of as fusion proteins that consist of two tandem TBT-bp2 repeat units [6,8]. Typical molecular masses of the protein components of TBT-bp2 were determined to be 21–23 kDa, which are almost half of that of PSTBP, based on the reported cDNA sequences that code this protein from several *Takifugu* species [8]. Recently, Satone et al. [9] functionally expressed PSTBP1 and 2 from *T. rubripes* in silkworm and found that both recombinant PSTBP1 and 2 have binding abilities for tributyltin, but only PSTBP2 has an affinity for TTX.

Hashiguchi et al. [8] examined the presence of cDNA that code TBT-bp2 and PSTBP in many species of fish by reverse transcription-polymerase chain reaction (RT-PCR) using degenerate primers and reported that more than one copy of each of the TBT-bp2 and PSTBP genes are present in five species of pufferfish in the genus *Takifugu*. They also identified TBT-bp2 genes in genera other than *Takifugu*, but to date, the PSTBP gene has not been found in pufferfish in genera other than *Takifugu* [8]. However, there are toxic pufferfish that belong to the genera *Arothron*, *Chelonodon*, *Tetraodon*, *Lagocephalus*, *Canthigaster*, and *Pleuranacanthus*, in addition to *Takifugu* [10]. We predict proteins similar to PSTBP in these genera, if PSTBP is implicated in the accumulation of TTX in toxic pufferfish as we proposed previously [1,4,5]. Therefore, we decided to examine the plasma of toxic pufferfish outside the genus *Takifugu* for the presence of PSTBP using analytical methods for protein. In this study, we investigated the presence of PSTBP-like proteins in the plasma of the toxic pufferfish *Arothron nigropunctatus*, *A. hispidus*, *A. manilensis* [11,12], and *Chelonodon patoca* [13], collected in Okinawa, Japan.

## 2. Results

### 2.1. TTX and Its Analogues in Pufferfish Plasma

**Figure 1:**
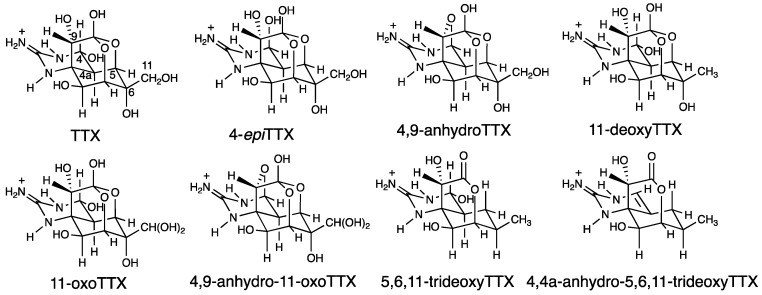
The structures of tetrodotoxin (TTX) and its analogues.

**Figure 2:**
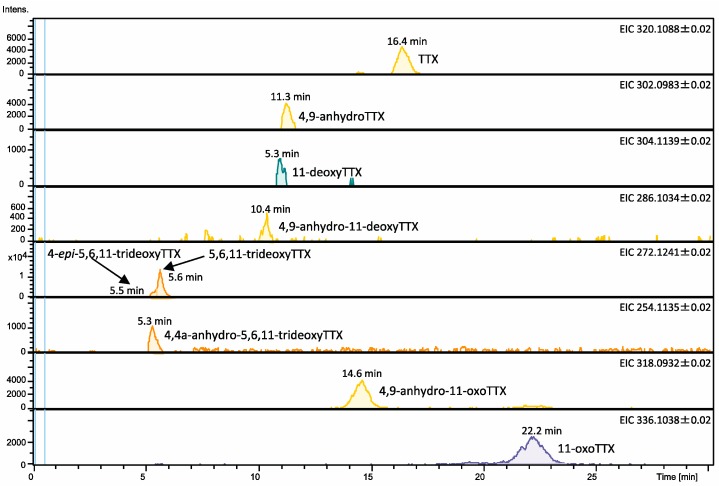
Liquid chromatography-mass spectrometry (LC-MS) chromatograms (EICs) of TTX and its analogues bound to the plasma proteins of *A. manilensis*, which were analyzed in their unbound forms following dissociation from the proteins fraction under acidic conditions. Calculated amounts of TTXs on the column: TTX (0.16 ng), 4,9-anhydroTTX (0.10 ng), 11-deoxyTTX (0.01 ng), 4,9-anhydro-11-deoxyTTX (0.01 ng), 4-*epi*-5,6,11-trideoxyTTX (0.07 ng), 5,6,11-trideoxyTTX (0.27 ng), 4,4a-anhydro-5,6,11-trideoxyTTX (0.02 ng), 4,9-anhydro-11-oxoTTX (0.19 ng), and 11-oxoTTX (0.17 ng). LC-MS conditions are same as previously reported [18].

We first confirmed that TTX and its analogues [14,15] (Figure 1) are present in their protein-bound forms in the blood plasma of *Arothron nigropunctatus*, *A. hispidus*, *A. manilensis*, and *C. patoca*. The high-molecular-weight compounds in the plasma were collected on an ultrafiltration filter (MW 30,000), after which the TTXs were dissociated from the proteins by treatment of the ultrafiltration filter with aqueous acetic acid solution (see Materials and Methods section for details). The dissociated TTXs were analyzed by liquid chromatography-fluorescence detection (LC-FLD) [16,17] and liquid chromatography-mass spectrometry (LC-MS) [18]. TTXs both in unbound and protein-bound forms were quantified, after which the total TTXs in the plasma were calculated. The results are summarized in Table 1, and the LC-MS chromatograms of the TTXs dissociated from the high-molecular-weight compounds in the plasma of *A. manilensis* are shown in Figure 2, as representatives. TTX, 4-*epi*TTX, 4,9-anhydroTTX, 5,6,11-trideoxyTTX, 4-*epi*-5,6,11-trideoxyTTX, and 4,4a-anhydro-trideoxyTTX were detected in the plasma of all species examined in this study. It is worth noting that 11-oxoTTX and 4,9-anhydro-11-oxoTTX were detected in relatively high concentrations in the plasma of *A. manilensis*.

### 2.2. The Ratios of TTXs Bound to Proteins to Total TTXs in Pufferfish Plasma

The ratios of TTXs bound to proteins to the total TTXs in pufferfish plasma were determined by ultrafiltration, LC-FLD, and LC-MS, and are summarized in Table 2. TTX and 5,6,11-trideoxyTTX-type analogues in all species examined appear to be present partially in protein-bound forms, with the protein-bound ratios of 5,6,11-trideoxyTTX and 4,4a-anhydro-5,6,11-trideoxyTTX higher than that of TTX. The ratios of bound/unbound TTXs determined by ultrafiltration do not directly reflect the affinity of each TTX analogue to plasma protein; the ratios can be influenced by the concentration of each analogue, and the equilibrium might be displaced by the separation of the free ligand depending on the binding affinity in the ultrafiltration method. However, at the least, this data clearly demonstrates that TTXs partially bind to high-molecular-weight compounds in pufferfish plasma of the three *Arothron* species and *C. patoca*, similar to those in the *Takifugu* species, in which binding of TTXs to high-molecular-weight compounds was previously indicated using the same ultrafiltration method [1,2,3].

### 2.3. Western Blot Analyses of Pufferfish Plasma with Anti-PSTBP Antibody Detection

Intact and deglycosylated pufferfish plasma following reaction with glycopeptidase F were analyzed for the presence of PSTBP-like proteins using Western blots with the anti-PSTBP antibody (anti-deglycosylated PSTBP, IgG fraction) that we had previously prepared [4]. We first confirmed that the plasma from two or three specimens of each species of *A. nigropunctatus*, *A. hispidus*, *A. manilensis*, and *C. patoca* exhibited the same species-specific bands, after which the plasma from one specimen of each species was examined by Western blot analysis.

**Figure 3:**
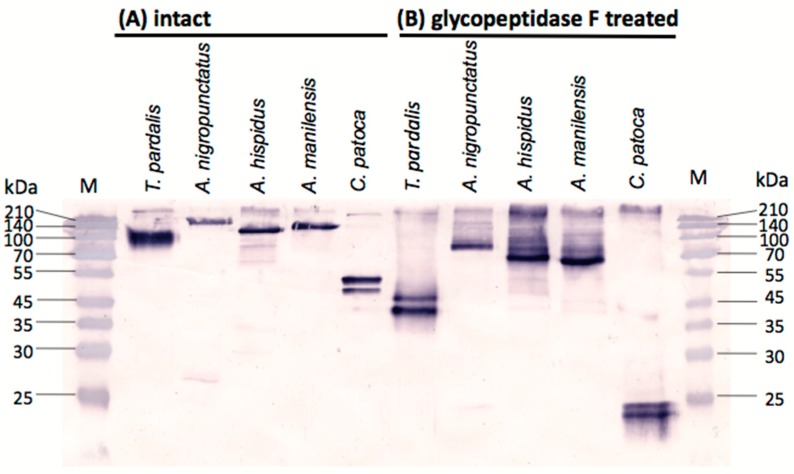
Western blot analysis (15% SDS-PAGE separation gel) of (**A**) intact plasma from five species of pufferfish (1 µg protein per lane); and (**B**) those after treatment with glycopeptidase F (1 µg protein per lane), detected with anti-pufferfish saxitoxin and tetrodotoxin binding protein (PSTBP) IgG.

As shown in Figure 3A, one or two major bands were detected in the intact plasma of each pufferfish species examined. SDS-PAGE indicated that the molecular masses of the detected bands for the *Arothron* species, namely *A. nigropunctatus*, *A. hispidus*, and *A. manilensis* were approximately 163 kDa, 118 kDa, and 130 kDa, respectively. These are larger than that of PSTBP found in *Takifugu pardalis* (108 kDa as the monomer) [2]. After treatment with glycopeptidase F (Figure 3B), the molecular masses of these bands for *A. nigropunctatus*, *A. hispidus*, and *A. manilensis* decreased to approximately 86 kDa, 71 kDa, and 67 kDa, respectively, which were also larger than that of the *Takifugu* PSTBP (43 kDa). Two bands were detected in the intact plasma of *C. patoca* (Figure 3A), the molecular masses of approximately 48 kDa and 50 kDa; their sizes are similar to that TBT-bp2 (48 kDa) found in Japanese flounder (*Paralichthys olivaceus*) [6]. In addition, the molecular masses of the two bands detected in the glycopeptidase F-treated plasma of *C. patoca* were observed to be approximately 22 kDa and 23 kDa, which are also similar to the predicted molecular masses of the protein component of TBT-bp2 (23 kDa) of Japanese flounder (*P. olivaceus*) [6] and the TBT-bp2 of the pufferfish of the genus *Takifugu* [8]. However, a band corresponding to TBT-bp2 (predicted molecular mass is 21 kDa) was not observed in the Western blot of *T. pardalis* (Figure 3B), even though the presence of cDNA-coded TBT-bp2 has been reported for this species [8]; this is probably because the antibody used against PSTBP in this study is unreactive toward TBT-bp2. This result suggested that the TBT-bp2-like proteins detected in *C. patoca* are more homologous with PSTBP than TBT-bp2, although the molecular masses of these proteins are similar to that of TBT-bp2.

### 2.4. N-Terminal Amino Acid Sequences of PSTBP-Like Proteins

**Figure 4:**
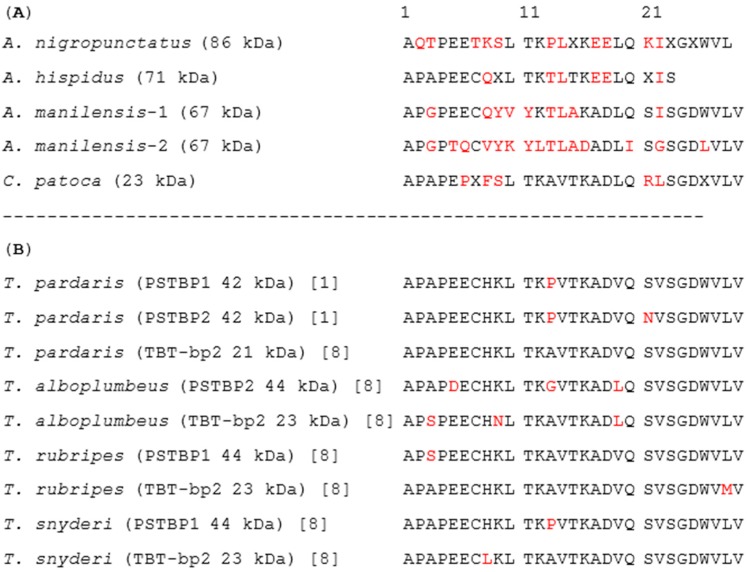
(**A**) Alignments of deduced *N*-terminal amino acid sequences of PSTBP-like proteins from the intact blood plasma of *A. nigropunctatus*, *A. manilensis*, *A. hispidus*, and *C. patoca* examined in this study with the molecular masses of their protein components; and (**B**) *N*-terminal amino acid sequences of the reported PSTBP and TBT-bp2 in *Takifugu* spp. with the molecular masses of their protein components deduced by cDNA sequences [1,8]. X denotes an amino acid that was not determined. The amino acid residues different from the major one are colored in red.

The major proteins in the intact plasma of each species of pufferfish that were detected by Western blot analysis with anti-PSTBP IgG (Figure 3A) were mostly purified by ammonium sulfate precipitation and HiTrap DEAE FF column chromatography, after which they were transferred to polyvinylidene difluoride (PVDF) membranes by electroblotting in order to examine their *N*-terminal amino acid sequences. Protein bands at approximately 163 kDa, 118 kDa, and 130 kDa were analyzed, for *A. nigropunctatus*, *A. hispidus*, and *A. manilensis*, respectively, while the band at 50 kDa was analyzed in the case of *C. patoca*. The first 23–29 residues of each *N*-terminal amino acid sequence were determined by Edman degradation on a gas-phase protein sequencer (Figure 4A). All determined N-terminal amino acid sequences were homologically similar to PSTBP and TBT-bp2, which are indistinguishable by their sequences in this region. In the case of *A. manilensis*, two amino acids were detected in some residues; while better sequence homology with PSTBP and TBT-bp2 was exhibited by *A. manilensis*-1, a different amino acid sequence was exhibited by *A. manilensis*-2 in Figure 4A. The result shown in Figure 4 suggests that PSTBP-like proteins are also present in the pufferfish of the three *Arothron* species examined, although the molecular masses of their deglycosylated proteins were larger than that of the PSTBP detected in the *Takifugu* species (Figure 3B) [4]. In addition, the proteins detected in *C. patoca* by Western blot analysis are also believed to be PSTBP-like proteins, because they were recognized by the anti-PSTBP antibody [4] which does not recognize TBT-bp2 [6,8] of *T. pardalis* (Figure 3) as we described above.

## 3. Discussion

According to many reports, TTX is produced by microorganisms [10,19,20] and then accumulated in pufferfish in high concentrations through the food chain [21,22]. TTX-binding proteins found in pufferfish are thought to play important roles in toxin accumulation and transport through pufferfish tissue, because several reports have suggested that pufferfish have the ability to accumulate TTX in their tissue, and this accumulated TTX partially moves to other tissues. For example, Kono et al. [23] reported that dietary TTX administered to cultured juvenile *Takifugu alboplumbeus* was accumulated in the liver and then gradually moved to the skin. Tatsuno and Arakawa et al. [24,25] reported that accumulated TTX in the liver is transferred to pufferfish ovaries depending on its stage of development and maturation. We found PSTBP in the pufferfish of the genus *Takifugu*, and proposed it is implicated in accumulation of TTX [1,4]. As a toxin-binding protein in the ovaries of *T. pardalis*, Yin and Nagashima et al. identified vitellogenin subdomain, a von Willebrand factor type D domain [26]. In addition, Nagashima et al. recently reported that *T. rubripes*, a toxic species of pufferfish, and non-toxic species such as *L. spadiceus*, *L. cheesemanii* and *S. pachygaster*, potentially absorb TTX into the liver, while non-toxic boxfish and porcupinefish do not take up either TTX or STXs [27].

In this study, the presence of PSTBP-like proteins in the plasma of three toxic *Arothron* species and *C. patoca* was confirmed for the first time using analytical methods for protein. This indicates that PSTBP is not a protein specific to the genus *Takifugu*, but is distributed over other genera of toxic pufferfish. It supports our hypothesis that PSTBP plays a role in the accumulation of TTX in toxic pufferfish belonging to a wide range of genera. In addition, we had previously confirmed that PSTBP-like protein was not detected by Western blot analysis with anti-PSTBP antibody in some non-toxic fish other than pufferfish [4]. Hashiguchi et al. [8] reported that the PSTBP gene was not found in the toxic pufferfish *Tetraodon nigroviridis*, but the cDNA of TBT-bp2, a homologous protein to PSTBP, was detected in this species and also in non-toxic pufferfish such as *Sphoeroides pachygaster*, *Lagocephalus wheelen*, and *L. gloveri*. We speculate that a few amino acid residue replacements in TBT-bp2 are responsible for determining their toxin-accumulating ability. Determining the full amino acid sequences of the PSTBP-like proteins and the TBT-bp2-like proteins found in this study will be the focus of future work.

## 4. Materials and Methods

### 4.1. Materials

The pufferfish used in the present study were all grown in the natural environment. *Takifugu pardalis* (12 male and one female, March 2001, total weight 5.5 kg) were purchased in Sendai, Japan. *A. nigropunctatus*, *A. hispidus*, *A. manilensis*, and *C. patoca* were collected in Okinawa, Japan, and were transported live to Sendai, Japan. The collection month, year, bodyweight, length, and gender were as follows: *A. nigropunctatus* (three specimens: April 2015, 118 g, 20 cm, male; April 2015, 70 g, 14 cm, male; November 2015, 134 g, 17 cm, female), *A. hispidus* (three specimens: May 2015, 112 g, 17 cm, male; June 2015, 124 g, 17 cm, male; April 2015, 697 g, 30 cm, female), *A. manilensis* (two specimens: November 2015, 332 g, 25 cm, male; December 2015, 372 g, 25 cm, female), and *C. patoca* (three specimens: May 2015, 209 g, 24 cm, female; May 2015, 77 g, 15 cm, female; November 2015, 185 g, 22 cm, female). The plasmas of the underlined specimens were used for TTXs analysis (Table 1 and Table 2), Western blot (Figure 3) and *N*-terminal amino acid sequence analysis (Figure 4) as representatives. The other specimens were used only for the preliminary Western blot to confirm that all specimens of each species showed species-specific bands. Blood plasma was collected from the portal vein of each live-fish specimen using a syringe precoated with sodium heparin (1000 U/mL). The plasma was kept in ice for a few minutes and then centrifuged for 30 min at 720× *g* at 4 °C, as reported previously [1]. These plasmas were kept at −80 °C before use. The protein concentrations were determined by the Lowry method using a DC protein assay kit (Bio-Rad, Hercules, CA, USA).

### 4.2. Quantitation of the TTXs in Pufferfish Plasma

The supernatant of the plasma (50 µL) from one specimen of each species of pufferfish was subjected to centrifugation at 15,000× *g* at 4 °C for 10 min, and then ultrafiltered using UltraFree MC (5000× *g*, 4 °C, 10 min × 2, 30,000 MW, Merck Millipore, Darmstadt, Germany) [3]. The filtrate was collected as the unbound TTX fraction. After the membrane was subjected to centrifugation with water (50 µL) as described above, the wash fraction was removed, and the TTX bound to high-molecular-weight compounds was dissociated by addition of 0.2 M acetic acid (50 µL) to the membrane, after which the filtrate was collected by centrifugation as described above for the bound fraction. TTXs in each fraction were quantified by LC-fluorescence detection [16,17] and LC-MS [18], following treatment with charcoal.

### 4.3. Reactions of Plasma with Glycopeptidase F

The plasma from each specimen (1 µg protein) in 10 µL of PBS was denatured by the addition of 5 µL of 0.5% (*w*/*v*) SDS/0.5 M Tris HCl (pH 8.6) containing 0.75% (*v*/*v*) 2-mercaptoethanol and heating for 3 min at 100 °C, and then reacted with 0.5 µU of glycopeptidase F (Takara Bio., Shiga, Japan) for 20 h at 37 °C in the presence of 1% (*w*/*v*) Nonidat P-40 in an aqueous solution with a total of 12.5 µL [4].

### 4.4. Western Blot Analysis

Western blot analyses of intact and glycopeptidase F-treated plasma from each pufferfish specimen were performed as reported previously by us [4]. Briefly, the plasma (1 µg protein) was denatured with 3 × SDS sample buffer and DTT (New England BioLabs, Ipswich, MA, USA) by heating at 95 °C for 5 min, separating on a 15% SDS-polycacrylamide gel [28], and transferring onto a nitrocellulose membrane (Bio-Rad, Hercules, CA, USA) in transfer buffer (25 mM Tris, 192 mM glycin, 20% methanol) according to the previously reported method [29]. After blocking the solution with PTB [1% (*w*/*v*) bovine serum albumin (BSA) in PT (1× PBS/0.1% (*v*/*v*) Tween-20)] for 1 h at room temperature, the membrane was incubated with anti-recombinant PSTBP IgG (1:1000) [4] diluted with PTB (5 mL) and allowed to stand at room temperature for 1 h. After washing three times with PT for 10 min, the secondary antibody, the anti-rabbit IgG-whole molecule alkaline phosphatase conjugate [Sigma-Aldrich Cat#A9919, 1:1000 diluted in PTB : goat serum (Gibco, Thermo Fisher Scientific, Waltham, MA, USA) (4:1, *v*/*v*)] was applied at room temperature for 1 h. The membrane was then washed three times with PT for 10 min, and the blots were developed with 1.3 mM nitrotetrazolium blue chloride (Sigma-Aldrich, St. Louis, MO, USA) and 1.3 mM 5-bromo-4-chloro-3-indoyl phosphate *p*-toluidine salt (Sigma-Aldrich) in 0.1 M Tris HCl pH 9.3 containing 0.1 M NaCl and 5 mM MgCl_2_. The reaction was terminated by the addition of 0.5 M EDTA (pH 8.0).

### 4.5. N-Terminal Amino Acid Sequences Analysis

The plasma proteins from each pufferfish were fractionated by precipitation with 30, 50, and 70% (*w*/*w*) ammonium sulfate. The bands were mainly detected in the supernatant of the 70% ammonium sulfate fraction by Western blot analysis with the anti-PSTBP antibody, for all species examined, which is similar to that observed for PSTBP [1]. The supernatant fraction was desalted by ultrafiltration (Amicon Ultra-0.5, 30,000 NMWL, Merck Millipore), and dissolved in 1 mL of PBS, after which it was applied to a HiTrap DEAE FF column (1 mL, GE Healthcare, Chicago, IL, USA) equilibrated with 50 mM Tris HCl (pH 7.4). PSTBP-like proteins were adsorbed to this column, then, they were eluted with 50 mM Tris HCl (pH 7.4) containing 0.1 M NaCl. These PSTBP-like proteins were further separated by SDS-PAGE (10% separating gel for *C. patoca*, 5% gel for *A. nigropunctatus*, *A. hispidus*, and *A. manilensis*), and then transferred onto PVDF membranes (Sequi-Blot^TM^ PVDF membrane, Bio-Rad) by electroblotting at 60 V and 500 mA for 180 min, after which they were stained with Coomassie Brilliant Blue R-250 (Sigma-Aldrich). The corresponding protein bands were carefully cut from the membranes, and washed thoroughly with ethanol and purified water. Each small piece of PVDF membrane, representing a protein band, was subjected to *N*-terminal amino acid sequencing with a gas-phase protein sequencer (PPSQ-10, Shimadzu, Kyoto, Japan) [30].

## 5. Conclusions

We investigated the presence of PSTBP analogues in the toxic pufferfish other than the genus *Takifugu*, three species in the genus *Arothron* and *Cheonodon patoca*. In the blood plasma of these species, TTX and its analogues were confirmed to be partially bound to high-molecular-weight compounds, similarly to *Takifugu* species. In addition, one or two bands were detected in the plasma of these four species by Western blot analysis with the anti-PSTBP antibody, but the molecular masses of their protein components were different from that of PSTBP. The *N*-terminal amino acid sequences of the detected glycoproteins were confirmed to be homologous with those of PSTBP and TBT-bp2 in this region. The results demonstrated that PSTBP is not a protein specific to the genus *Takifugu*, but is distributed over other genera of toxic pufferfish; these results support our hypothesis that PSTBP and its analogues are responsible for the accumulation of TTX in toxic pufferfish.

## Figures and Tables

**Table 1:** The total TTX concentrations in pufferfish plasma (ng/mL).

	*A. nigropunctatus*	*A. hispidus*	*A. manilensis*	*C. patoca*
TTX	150	91	890	1000
4-*epi*TTX	78	12	60	110
4,9-anhydroTTX	19	15	850	95
11-oxoTTX	45	ND	1100	28
4,9-anhydro-11-oxoTTX	96	ND	2800	ND
11-deoxyTTX	120	ND	74	170
5,6,11-trideoxyTTX	95	22	810	59
4,4a-anhydro-trideoxyTTX	100	32	75	30

The sums of the unbound and bound forms of TTXs in the plasma of one specimen of each species were quantified by liquid chromatography-fluorescence detection (LC-FLD) and/or LC-MS. ND denotes not detected (less than the limit of detection (LOD): *s*/*n* > 5, 14 ng/mL).

**Table 2:** The ratios (mol %) of protein-bound TTXs to the total TTXs in pufferfish plasma.

	*A. nigropunctatus*	*A. hispidus*	*A. manilensis*	*C. patoca*
TTX	32	26	53	12
4-*epi*TTX	ND	ND	ND	17
4,9-anhydroTTX	ND	ND	51	ND
11-oxoTTX	68	ND	70	ND
4,9-anhydro-11-oxoTTX	ND	ND	61	ND
11-deoxyTTX	14	ND	29	3
5,6,11-trideoxyTTX	66	72	75	100
4,4a-anhydro-5,6,11-trideoxyTTX	76	82	85	100

Ratios were determined by ultrafiltration. Unbound TTXs were quantified by LC-FLD and/or LC-MS. ND indicates that TTXs were not detected in the protein-bound fraction (less than LOD: *s*/*n* > 5, 14 ng/mL).
